# Prognostic parameters of luminal A and luminal B intrinsic breast cancer subtypes of Pakistani patients

**DOI:** 10.1186/s12957-017-1299-9

**Published:** 2018-01-02

**Authors:** Atif Ali Hashmi, Saher Aijaz, Saadia Mehmood Khan, Raeesa Mahboob, Muhammad Irfan, Narisa Iftikhar Zafar, Mariam Nisar, Maham Siddiqui, Muhammad Muzzammil Edhi, Naveen Faridi, Amir Khan

**Affiliations:** 1 Liaquat National Hospital and Medical College, Karachi, Pakistan; 2 Shaheed Zulfiqar Ali Bhutto Institute of Science and Technology, Karachi, Pakistan; 3 Aga Khan University, Karachi, Pakistan; 4 Department of Pathology, Liaquat National Hospital and Medical College, Karachi, Pakistan; 5 Department of Surgery, Brown University, Providence, RI USA; 6 Department of Medicine, Kandahar University, Kandahar, Afghanistan

**Keywords:** Luminal A, Luminal B, ER, PR, Her2neu, Breast cancer

## Abstract

**Background:**

Prognosis of breast cancer and success of therapeutic interventions largely rely on the clinico-pathologic and biological characteristics of the tumor and vary due to the heterogeneous nature of breast cancers. The aim of this study was to determine the frequency and prognostic parameters of luminal breast cancers in our population to devise targeted and personalized therapeutic regimens tailored to the needs of the loco-regional population.

**Methods:**

A retrospective cross-sectional study including 1951 cases of primary breast cancer treated at Liaquat National Hospital Karachi was conducted during the year 2011–2016. The clinico-pathologic characteristics were observed and semiquantitative immunohistochemical analysis was performed to study the luminal subtypes A and B. The cross-tabulated statistics of the observed characteristics were performed between the two subtypes. The significance level of each characteristic was estimated utilizing the chi-square test.

**Results:**

Luminal cancers comprised 62.7% of the total number of cases diagnosed with breast cancers in the study period. Out of these 1224 cases of luminal cancers, 845 cases (69%) were luminal B, while 379 (31%) cases were of luminal A. Luminal B cancers were significantly more common in younger age groups as compared to luminal A cancers. Comparison of the two subtypes of luminal breast cancers revealed significant differences. Luminal B cancers were associated with higher grade (26% grade III in luminal B compared to 8% in luminal A), micropapillary histology, and high frequency of nodal metastasis (54 vs. 43%).

**Conclusions:**

Luminal B comprised the most frequent subtype of breast cancer in our study and they were found more constantly in a younger age group. Moreover, they were associated with adverse clinico-histologic parameters like higher grade and nodal metastasis. Therefore, we suggest that, despite lack of widespread availability of molecular studies in our setup, IHC-based typing should be done in every case of breast cancer to individualize therapy.

## Background

Luminal breast cancers were first defined by molecular profiling as tumors with gene expression similar to the luminal epithelium of the breast. Luminal A tumors are marked by high expression of estrogen receptor(ER)-related genes, low expression of human epidermal growth factor receptor 2 (HER2neu) genes and low expression of proliferation-related genes [1, 2]. On the other hand, luminal B tumors exhibit low expression of ER-related genes, variable expression of HER2neu-related genes, and high expression of proliferation-related genes with worse prognosis than luminal A tumors [3]. St. Gallen International Expert Consensus on the “Primary Therapy of Early Breast Cancer 2013” proposed that luminal subtypes of breast cancer can be defined in view of immunohistochemistry (IHC) for ER, progestrone receptor (PR), HER2neu, and ki67 index rather than conventional molecular diagnostics [4]. Ki67 index reflects the proportion of the proliferation genes/cells and thus employed to subtype luminal tumors. Therefore, Ki67 index is also utilized to tailor the therapy for the patient as in some cases chemotherapy or endocrine therapy alone is inadequate [5]. Although exact cutoffs for ki67 and PR were not defined in St. Gallen International Expert Consensus, however, some proposals were given, stating that a cutoff value of < 14% ki67 can be used to define luminal cancers in the absence of HER2neu expression based on single reference laboratory [6]. On the other hand, Prat et al. expressed that a cutoff value of > 20% can be adapted to ascertain luminal A cancers. These proposals were derived from the results of five cohorts, which were combined and interpreted by experts [7]. It is also well established that enormous heterogeneity exists in genetic and morphological characteristics of breast tumors. On account of this heterogeneity, therapeutic regimens must be tailored to the loco-regional breast cancer profiles. Studies have demonstrated a different behavior of breast cancer in Pakistan with higher prevalence in a younger age group with aggressive nature and poor survival rates [8–10]. Therefore, we aimed to determine prognostic parameters of luminal breast cancers in our population which can help in devising targeted and personalized therapeutic regimens tailored to the needs of the loco-regional population.

## Methods

It was a retrospective cross-sectional study including 1951 cases of primary breast cancer treated at Liaquat National Hospital Karachi from the year 2011 till 2016. An approval from an ethical review committee was taken antecedent to conducting the study. Specimens included trucut biopsies, wide local excisions + lymph node dissection, or simple/modified radical mastectomies based on sentinel lymph node status. Histopathologic characteristics recorded include tumor type, size, grade, and lymph node status along with degree of necrosis, lymphocytic reaction, and fibrosis. Lymphocytic infiltration (tumor infiltrating lymphocytes) was assessed in the stromal compartment of the tumor. Percentage of stromal area showing dense mononuclear infiltrate was evaluated and scored as a continuous variable. However, as no clinically relevant threshold was defined yet, therefore, we took cutoffs of < 10% as mild to moderate and > 50% as severe lymphocytic infiltration [11]. Lymphovascular invasion was assessed outside the borders of invasive carcinoma. The tumor emboli should not exactly conform to the contours of the space in which they were found. In addition, endothelial cell nuclei should also be identified [12]. One representative section is selected for IHC studies including ER, PR, her2neu, and ki67 by DAKO envision method.

The results for ER and PR were scored in a semiquantitative fashion incorporating both the intensity and the distribution of specific staining [13]. For each tissue, intensity and percentage of positive staining cells were determined. In addition, a value designated as H-SCORE was derived by summing up the percentage of cell staining multiplied by the weighted intensity of staining (negative—0, weak—1, moderate—2, strong—3) with 300 possible values (1–300) [14]. More than 1% staining cells was considered positive expression.

HER2neu were scored based on the intensity and percentage of positive cells on a scale of 0 to 3+ according to American Society of Clinical Oncology/College of American Pathologists (ASCO/CAP) guidelines. Cases were reported 0 (negative) if no staining observed or incomplete, faint/barely perceptible membrane staining in ≤ 10% of invasive tumor cells; 1+ (negative) if incomplete, faint/barely perceptible membrane staining in > 10% of invasive tumor cells; 2+ (positive) if incomplete and/or weak to moderate circumferential membrane staining in > 10% of invasive tumor cells or complete, intense, circumferential membrane staining in ≤ 10% of invasive tumor cells; or 3+ (positive) if complete, intense, circumferential membrane staining in > 10% of invasive tumor cells [15, 16]. Cases with intermediate (2+) expression of Her2neu underwent subsequent fluorescence in situ hybridization (FISH) testing for Her2neu gene amplification. FISH testing was performed using FDA-approved Path Vysion Her2 DNA Probe kit.

Results were expressed as the ratio of Her2 signals as compared to CEP 17 signals according to ASCO/CAP guidelines. A result of HER2/CEP17 ratio < 2.0 and average HER2 copy number < 4.0 signals/cell is considered negative (not amplified); HER2/CEP17 ratio < 2.0 and average HER2 copy number ≥ 4.0 but < 6.0 signals/cell as equivocal; and HER2/CEP17 ratio ≥ 2.0 (regardless of average HER2 copy number) or average HER2 copy number ≥ 6.0 signals/cell (regardless of ratio) as positive (amplified).

Ki-67 immunoreactivity was recorded as continuous variables, based on the proportion of positive tumor cells (0–100%). At least 1000 cells were evaluated before calculating an average estimate. If hot spots were present, then they are included in the estimation. Ki67 index was further categorized into four groups, < 14, 15–24, 25–44, and > 45%.

Luminal A and luminal B breast cancers were defined as follows:

Luminal A: ER and/or PR positive, ki67 low (< 14%).

Luminal B: ER and/or PR positive with ki67 high (> 14%) or ER and/or PR positive and Her2neu positive irrespective of ki67.

All cases of primary breast cancers which were classified as luminal according to the above criteria were included in the study. Cases of recurrent breast cancers and those with prior chemotherapy were excluded from the study.

Statistical package for social sciences (SPSS 21) was used for data compilation and analysis. Mean and standard deviation were calculated for quantitative variables. Frequency and percentage were calculated of qualitative variables. Shapiro-Wilk test was applied to test normality. Chi-square was applied to see association. Mann-Whitney test was applied to compare difference in means among groups by considering *P* value ≤ 0.05 as significant.

## Results

A total of 1951 cases of primary breast cancer diagnosed at our facility were assessed for inclusion and exclusion criteria. One thousand two hundred twenty-four cases were included in our study as they met the diagnostic criteria of luminal type cancers which were estrogen receptor and progesterone receptor positive. Luminal cancers comprised 62.7% of the total number of cases diagnosed with breast cancers. Out of these 1224 cases, 845 cases, i.e., 69% of the samples, were luminal B, while 379, i.e., 31% of the cases, were luminal A. Table 1 shows the comparison of these two categories of luminal cancers.

**Table 1:** Co-relation of clinico-pathologic parameters of luminal A and luminal B breast cancers

		Luminal B	Luminal A	Total	*P* value
Age (years) ↑±		51.12 ± 12.619	54.63 ± 12.740		< 0.05
Age group (*n* = 1224)					
	≤ 30	42(5)	14(3.7)	56(4.6)	< 0.05
	31–50	397(47)	138(36.4)	535(43.7)	
	51–70	355(42)	194(51.2)	549(44.9)	
	> 70	51(6)	33(8.7)	84(6.9)	
Tumor grade (*n* = 1224)					
	Grade 1	94(11.1)	112(29.6)	206(16.8)	< 0.05
	Grade 2	527(62.4)	235(62)	762(62.3)	
	Grade 3	224(26.5)	32(8.4)	256(20.9)	
Size of tumor ↑±		35.09 ± 14.20	35.21 ± 15.18		0.985
Tumor stage (*n* = 504)					
	T1	41(13.7)	35(17.2)	76(15.1)	0.535
	T2	216(72)	139(68.1)	355(70.4)	
	T3/T4	43(14.3)	30(14.7)	73(14.5)	
Nodal status (*n* = 504)					
	Positive	162(54)	89(43.6)	251(49.8)	< 0.05
	Negative	138(46)	115(56.4)	253(50.2)	
N stage (*n* = 504)					
	N0	141(47)	115(56.4)	256(50.8)	< 0.05
	N1	74(24.7)	50(24.5)	124(24.6)	
	N2	50(16.7)	16(7.8)	66(13.1)	
	N3	35(11.7)	23(11.3)	58(11.5)	
Laterality (*n* = 1224)					
	Left	419(49.6)	191(50.4)	610(49.8)	0.793
	Right	426(50.4)	188(49.6)	614(50.2)	

Chi-square was applied

±Mean ± SD

↑Mann-Whitney test

Mean age for luminal B cancers was found to be 51.1 years, which was significantly lower in relation with luminal A cancers for which the mean age was 54.6 years. Notably, luminal B cancers were of high grade as compared to luminal A cancers as the frequency of grade 3 tumors in luminal B was found to be 26.5%, while in luminal B, the frequency was only 8.4%. Similarly, nodal metastasis was more prominent in luminal B as 54% of luminal B cases had nodal metastasis and only 43% of luminal A had nodal metastasis (*P* value < 0.05).

As far as biomarker status is concerned, 73% of luminal cancers were PR positive as compared to 83% positivity in luminal A cancers. Table 2 shows comparison of histologic features of luminal A and B cancers. Lymphovascular invasion was seen more frequently in luminal B cancers compared to luminal A cancers.

**Table 2:** Co-relation of histologic features of luminal A and luminal B breast cancers. Chi-square was applied

		Luminal B	Luminal A	Total	*P* value
Lymphocytic infiltration (*n* = 504)					
	Absent	197(65.7)	151(74)	348(69)	0.060
	Mild to moderate	85(28.3)	48(23.5)	133(26.4)	
	Severe	18(6)	5(2.5)	23(4.6)	
In situ component (*n* = 503)					
	Present	214(71.6)	155(76)	369(73.4)	0.272
	Absent	85(28.4)	49(24)	134(26.6)	
Lymphovascular invasion (*n* = 504)					
	Present	88(29.3)	31(15.2)	119(23.6)	≤ 0.05
	Absent	212(70.7)	173(84.8)	385(76.4)	
Dermal lymphatic invasion (*n* = 504)					
	Present	38(12.7)	30(14.7)	68(13.5)	0.511
	Absent	262(87.3)	174(85.3)	436(86.5)	
Pagetoid spread (*n* = 504)					
	Present	5(1.7)	2(1)	7(1.4)	0.707
	Absent	295(98.3)	202(99)	497(98.6)	

The histologic subtyping of the luminal A and luminal B cancers also showed significant variations. Micropapillary histology was noted more frequently in luminal B subtype while frequency of lobular, cribriform, papillary, and mucinous tumors was higher in luminal A subtype of breast cancers. Table 3 depicts the distribution of different histologic subtypes of breast cancer in these two categories of luminal cancers.

**Table 3:** Distribution of histological types of breast carcinoma among luminal A and luminal B breast carcinoma

	Luminal B	Luminal A	Total
IDC	747(88.4)	287(75.7)	1034(84.5)
ILC	41(4.9)	41(10.8)	82(6.7)
Cribriform	3(0.4)	4(1.1)	7(0.6)
Papillary	14(1.7)	18(4.7)	32(2.6)
Mucious	12(1.4)	22(5.8)	34(2.8)
Micropapillary	13(1.5)	0(0)	13(1.1)
Tubular	4(0.5)	6(1.6)	10(0.8)
Metaplastic	8(0.9)	1(0.3)	9(0.7)
Mixed	3(0.4)	0(0)	3(0.2)
Total	845	379	1224

## Discussion

In Asian and African regions, the incidence of breast cancer among women below the age of 40 years is 30%, which is significantly high as compared to the rest of the world [17–19]. Therefore, the present study investigated the prognostic parameters of luminal breast cancers in Pakistani population, to assist the physicians in tailoring targeted and personalized hormonal and chemotherapeutic interventions. Acknowledged by previous studies, gene expression profiling helps in identifying biologically distinct breast cancer subtypes which have exclusive prognosis [13, 20]. Understanding of the gene expression of breast cancer is notably important in determining the therapeutic interventions; however, the biological behavior of the molecular subtype is largely characterized by the clinico-pathologic features of the tumor [21]. The results of the present study revealed that 62.7% of primary breast cancers were luminal in nature. In our study, luminal B cancer was more prevalent (69%) than luminal A (31%). The results are in concordance with other studies conducted in Italy (34% luminal A and 36% luminal B) [22] and Saudi Arabia (3.9% luminal A and 16% luminal B) [23] which found luminal B subtypes more common than luminal A albeit with other studies conducted in various parts of the world including the USA (55% luminal A and 17% luminal B) [24], Tunis (51.5% luminal A and 16% luminal B) [25], Japan (71% luminal A and 8% luminal B) [26], Egypt (44.3% luminal A and 24.6% luminal B) [27], China (65.3% luminal A and 19% luminal B) [28], and Algeria (50.6% luminal A and 19.% luminal B) [29] which found luminal A as the most prevalent molecular subtype of breast cancer. The variation in the commonest profile of breast cancer explains the heterogeneity of breast cancer across the world.

Additionally, the clinico-pathologic parameters of luminal A and luminal B breast cancers including age, tumor size, grade, ki67 value, tumor stage, nodal status, and laterality were correlated. The frequency of luminal B subtype was notably higher in a younger age group as compared to luminal A, which was more frequent in older age. Clinically, luminal B cancers are associated with poor outcomes and aggressive behavior even in the presence of concomitant therapy [15]. The highest numbers of patients with luminal B subtype (47%) were between the age group of 31 to 50 years, making it the most widespread subtype of breast cancer among young-age women. These findings are in congruence with other studies that revealed that the incidence of breast cancer is considerably higher among premenopausal women (below 40 years) in low- and middle-income countries [30]. A similar study conducted in Turkey revealed that approximately half of the patients diagnosed with breast cancer were premenopausal, and 20% of them were below the age of 40 years [31].

Moreover, 26.5% of luminal B type cancers were high grade as compared to luminal A cancers. The present study also demonstrated that luminal B cancers were more advancing as more than 50% of the tumors exhibited nodal metastasis, while in luminal A subtype, only 43% tumors exhibited nodal metastasis. Apart from tumor grade, axillary involvement is another predominant prognostic factor of breast cancer [27, 32]. In our study, the rate of axillary positivity was more pronounced among younger patients. Alternately, tumor size is used to access prognosis of breast cancer in the absence of nodal involvement [23]. Apropos of tumor characteristics, the luminal B subtype was more aggressive in our study as nodal involvement; tumor grade were significantly high in luminal B. These findings are consistent with another research study which indicated that luminal B cancers are characterized by larger tumor size, extensive nodal involvement, and advanced tumor stage as compared to luminal A subtypes [33].

Lymphovascular invasion is another prognostic factor that determines the risk of recurrence after treatment [34]. Lymphovascular invasion was more frequent in luminal B cancers, which implies that post-treatment recurrence of disease in luminal B cancers make them more challenging to manage and also reduce the survival rate in these patients. The study also revealed a high frequency of lobular, cribriform, papillary, and mucinous characteristics in luminal A subtype of breast cancers while luminal B cancers were more aggressive in nature on account of advanced tumor grade, nodal involvement, lymphovascular invasion, and higher frequency of micropapillary histology. These findings are consistent with other studies which demonstrated that micropapillary carcinomas are more intrusive in nature due to their propensity to disseminate to axillary lymph nodes and these carcinomas are usually of luminal B type [35, 36]. Moreover, papillary and mucinous phenotypes are more indolent as compared to micropapillary phenotype, thus accounting for the more aggressive nature of luminal B subtypes.

The major limitation of this study is lack of follow-up/recurrence data; however, detailed histological and immunohistochemical features of a large series of breast cancer presented in our study will be useful in tailoring therapeutic regimens in loco-regional population.

## Conclusion

In conclusion, prognosis and treatment of breast cancer largely depends upon the tumor characteristics including histological features, genetic profile, and clinico-pathologic parameters of the tumor. So, we suggest that each healthcare facility must conduct detailed histological, genetic, and molecular subtyping before tailoring a standard treatment regimen for each patient. The results of our study also indicated that luminal B, which is an aggressive type of breast cancer, is more prevalent among younger patients, which demonstrate poor prognosis in younger age as compared to older age.
